# Loss of *Tmem30a* leads to photoreceptor degeneration

**DOI:** 10.1038/s41598-017-09506-5

**Published:** 2017-08-24

**Authors:** Lin Zhang, Yeming Yang, Shujin Li, Shanshan Zhang, Xiong Zhu, Zhengfu Tai, Mu Yang, Yuqing Liu, Xinzheng Guo, Bo Chen, Zhilin Jiang, Fang Lu, Xianjun Zhu

**Affiliations:** 1 Sichuan Provincial Key Laboratory for Human Disease Gene Study and School of Medicine, Hospital of the University of Electronic Science and Technology of China and Sichuan Provincial People’s Hospital, Chengdu, Sichuan 610072 China; 2 Department of Laboratory Medicine, Sichuan Academy of Medical Sciences and Sichuan Provincial People’s Hospital, Chengdu, Sichuan 610072 China; 3 Institute of Laboratory Animal Sciences, Sichuan Academy of Medical Sciences and Sichuan Provincial People’s Hospital, Chengdu, Sichuan China; 4 Chengdu Institute of Biology, Chinese Academy of Sciences Sichuan Translational Medicine Research Hospital, Chengdu, Sichuan China; 5 Department of Ophthalmology, Yale University School of Medicine, New Haven, CT USA

## Abstract

Phosphatidylserine (PS) is asymmetrically distributed between the outer and inner leaflets of the plasma membrane in eukaryotic cells. PS asymmetry on the plasma membrane depends on the activities of P4-ATPases, and disruption of PS distribution can lead to various disease conditions. Folding and transporting of P4-ATPases to their cellular destination requires the β subunit TMEM30A proteins. However, the *in vivo* functions of *Tmem30a* remain unknown. To this end, we generated retinal-specific *Tmem30a*-knockout mice to investigate its roles *in vivo* for the first time. Our data demonstrated that loss of *Tmem30a* in mouse cone cells leads to mislocalization of cone opsin, loss of photopic electroretinogram (ERG) responses and loss of cone cells. Mechanistically, *Tmem30a*-mutant mouse embryonic fibroblasts (MEFs) exhibited diminished PS flippase activity and increased exposure of PS on the cell surface. The broad loss of *Tmem30a* in adult mice led to a reduced scotopic photoresponse, mislocalization of ATP8A2 to the inner segment and cell body, and increased apoptosis in the retina. Our data demonstrated novel essential roles of *Tmem30a* in the retina.

## Introduction

Phospholipids are asymmetrically distributed between the outer and inner leaflets of the plasma membrane in eukaryotes^1, 2^. Phosphatidylserine (PS) is primarily located in the inner cytoplasmic leaflet. PS flippases, which belong to the P4-ATPase family, transport aminophospholipids from the exoplasmic to the cytoplasmic leaflet of cell membranes by utilizing ATP^3–6^. Aminophospholipid asymmetry in the cellular membrane that is maintained by P4-ATPases is critical for various biological processes, such as blood coagulation regulation^7^, vesicular protein transport^8^, the recognition of apoptotic cells^9^ and sperm capacitation^10^.

The fact that mutations in several P4-ATPases, including ATP8B1, ATP8A2 and ATP11C, lead to various human diseases highlights the importance of P4-ATPases^11–16^. For instance, mutations in *ATP8B1* cause progressive familial intrahepatic cholestasis type I and benign recurrent intrahepatic cholestasis^11^, and mutations in *ATP8A2* cause axonal degeneration in mice and a severe neurological disorder that is characterized by cerebellar ataxia, mental retardation and disequilibrium syndrome^12, 14–16^. Mice deficient in *Atp8a1* display increased externalization of PS on the plasma membranes of hippocampal cells and a deficiency in hippocampus-dependent learning^13^. In addition, *Atp10c* is associated with Angelman syndrome^17^, and *Atp11c*-deficient mice have recently been reported to exhibit B-cell lymphopenia, anaemia and intrahepatic cholestasis^18–21^. During apoptosis, ATP11C is also inactivated by caspases^22^.

P4-ATPases, except ATP9A and ATP9B, are associated with members of the conserved CDC50 protein family^23^. There are three CDC50 genes in the mammalian genome (*CDC50A*, *CDC50B* and *CDC50C*, also named *TMEM30A*, *TMEM30B* and *TMEM30C*, respectively), which encode N-glycosylated proteins with two transmembrane segments^23^. Emerging evidence indicates that TMEM30 proteins are essential for the folding and exporting of P4-ATPases from the endoplasmic reticulum^24–29^. TMEM30 proteins also play roles in the catalytic reaction cycle of P4-ATPase^23, 29^. Akin to the beta subunit of the Na+/K+ ATPase, TMEM30 proteins act as the beta-subunit of P4-ATPase and participate in ATP-dependent phospholipid transport^23, 29, 30^. In the retina, TMEM30A was expressed in photoreceptor cells and is critical for the stable expression, transport and lipid transport activity of the P4-ATPase ATP8A2, as determined by heterologous expression studies and *in vitro* analysis^31^. In the retina, *Atp8a2* is expressed in photoreceptor cell and essential for retinal photoreceptor function and survival^32^. Loss of ATP8A2 in either *Atp8a2* mutant *wl* or *vmd*, or knockout mouse model led to reduced scotopic and photopic ERG responses, shorted outer segment, thinner outer nuclear layer and degeneration of rod cells^32^. However, the *in vivo* functions of *Tmem30a* in the mammalian retina are yet to be elucidated.

In this study, we generated the first retina-specific *Tmem30a*-knockout mice and investigated the *in vivo* functions of *Tmem30a* in mice. Our data showed that loss of *Tmem30a* in mouse cone cells led to the mislocalization of cone opsin protein, the loss of photopic electroretinogram (ERG) responses and the loss of cone cells. Broad deficiency of *Tmem30a* in adult mice causes a reduced scotopic photoresponse and the mislocalization of PS flippase ATP8A2 to the inner segment and cell body, which leads to the dysfunction and death of rod cells. The loss of *Tmem30a* in mouse embryonic fibroblasts (MEFs) resulted in reduced PS flippase activity and increased exposure of PS on the cell surface. Collectively, our data demonstrated that the loss of *Tmem30a* leads to mislocalization of PS flippase ATP8A2 and degeneration of retinal rod and cone cells. Thus, our studies highlight an essential role for *Tmem30a* in the retina.

## Results

### *Tmem30a* is essential for survival

**Figure 1:**
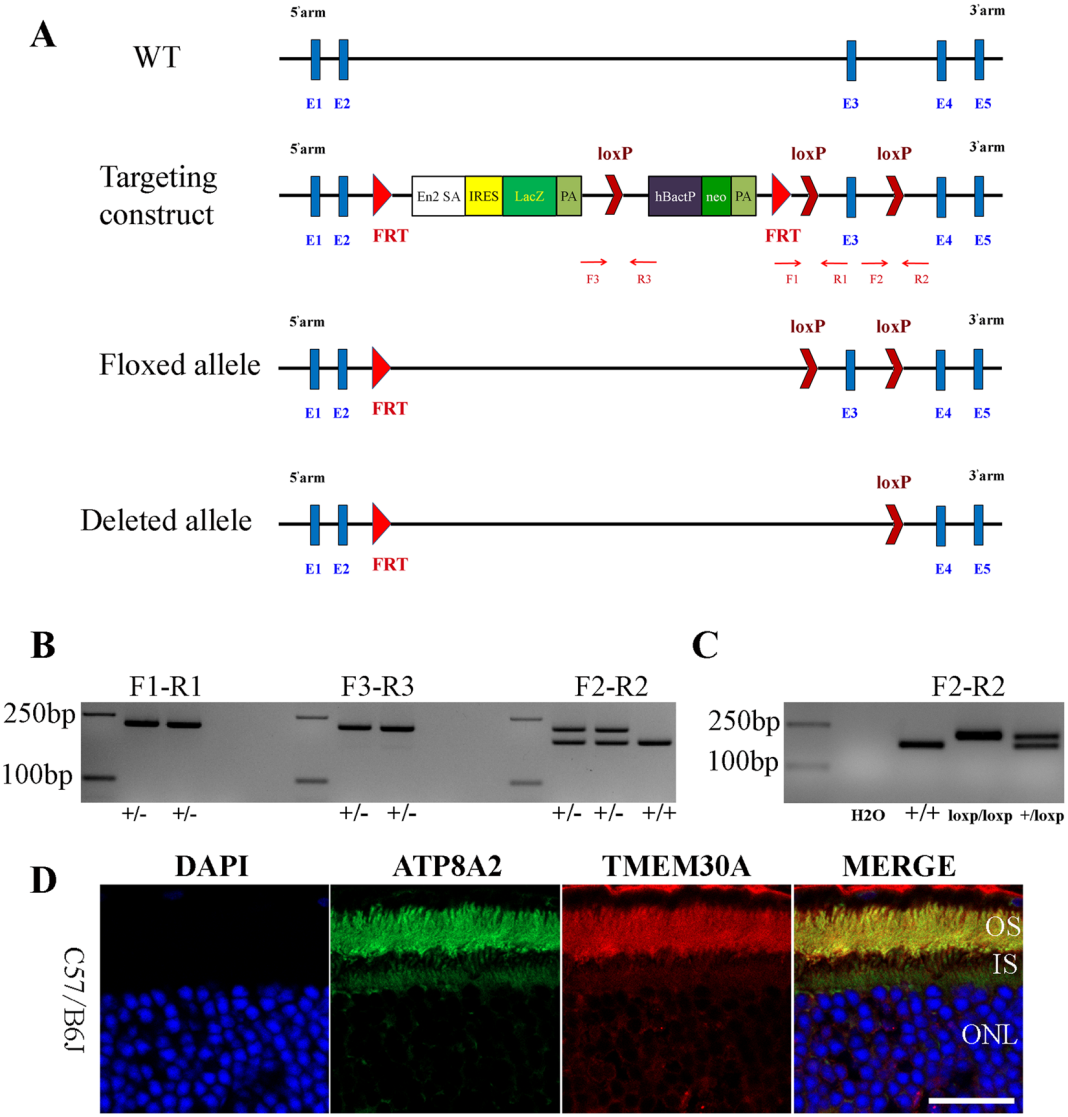
Generation of the *Tmem30a*-knockout-first allele. (**A**) Scheme showing the targeting strategy for the disruption of the *Tmem30a* gene. The knockout-first allele design is shown with the LacZ reporter. Exon 3 is flanked by two loxP sites. PCR primers used to genotype the target allele are shown beneath the diagram. Primer pair F1-R1 was designed to genotype the loxP site upstream of exon 3. Primer pair F2-R2 was designed to genotype the loxP site downstream of exon 3. Primer pair F3-R3 was designed to genotype the loxP site upstream of the human beta actin promoter (hBactP). After crossing with the Flper deletion line, the FRT-flanked reporter cassette was removed, resulting in a floxed allele. The critical exon (exon 3) is flanked by two loxP sequences. When the floxed allele was crossed to a Cre-expressing line, exon 3 was deleted, resulting in a frame-shifting deleted allele. (**B**) Genotyping of *Tmem30a*-knockout first mice. Using primer pair F1-R1, PCR amplification of genomic DNA extracted from mouse tails produced products of 220 bp in knockout mice. Using primer pair F2-R2, PCR amplification of genomic DNA extracted from mouse tails produced products of 214 bp in knockout mice and 179 bp in wild-type mice. Gel picture was cropped to save space. Full gel picture was listed as Figure S9. (**C**) Genotyping of *Tmem30a* conditional knockout mice. Using primer pair F2-R2, PCR amplification of genomic DNA from mouse tails produced products of 214 bp in homozygous conditional knockout mice and 179 bp in wild-type mice. Two products were amplified in heterozygous mice. Gel picture was cropped to save space. Full gel picture was listed as Figure S9. (**D**) Colocalization of TMEM30A with ATP8A2 in the mouse retina. Immunofluorescence staining of cryosections of retinas from wild-type mice at 30 days of age using a polyclonal antibody to ATP8A2 (green) and a monoclonal antibody to TMEM30A (red). Nuclei were counter stained with DAPI (blue). Both proteins mainly localized to the outer segment of the photoreceptor cells. Weak expression in the inner segment was observed. Scale bar: 25 μm.

*Tmem30a* is broadly expressed in the retina, brain, cerebellum, liver, heart, kidney, spine and testis (Figure S1). To investigate the role of *Tmem30a in vivo*, we generated *Tmem30a*-knockout mice (Fig. 1). In the modified *Tmem30a* allele, an FRT-flanked bacterial beta-Gal reporter gene with an upstream splicing acceptor and a neomycin expression cassette were inserted into intron 2–3. Exon 3 was flanked by two loxP sites (Fig. 1A). This constitutes a knockout allele with the potential to be converted into a conditional knockout allele. With a splicing acceptance site (SA) in place in intron 2–3, transcription of *Tmem30a* mRNA is disrupted, resulting in a null allele (*Tmem30a* ^*tm1Xjz*^). The knockout allele was confirmed by long range PCR: a 6.3-Kb fragment was amplified for the 5′arm and a 6.6-Kb fragment was amplified for the 3′arm (Figure S2). PCR genotyping was used to identify possible homozygous knockout mice from their wild-type and heterozygous littermates (Fig. 1B). Of 64 pups tested on P1, no homozygous mutant mice were observed (Table S1). Therefore, *Tmem30a* is essential for early embryonic development.

### The role of *Tmem30a* in the retina

In the retina, TMEM30A localized to the outer segment and inner segment (with weak expression) of photoreceptor cells, similar to ATP8A2^31, 32^ (Fig. 1D). The specificity of mouse monoclonal anti-TMEM30A antibody was verified (Figure S1B, C). To study the role of *Tmem30a* after early development, the *Tmem30a* knockout first allele was crossed to Flper mice^33^ to remove the FRT-flanked LacZ reporter and Neo cassette and to generate a conditional allele, *Tmem30a* ^*tm1.1Xjz*^. For simplicity, this allele is referred to as *Tmem30a* ^*loxp*^ (Fig. 1C). Pan-neural deletion of *Tmem30a* using Nestin-Cre^34^ led to early lethality (Table S2). To investigate the role of *Tmem30a* in the retina, we generated retinal neural deletion mutant mice using Six3-Cre^35^ (Figure S3A). *Tmem30a* expression was diminished to 25% in mutant retina (Figure S3A). Loss of *Tmem30a* in retinal neuronal progenitor cells resulted in the massive loss of neural cells and the degeneration of retina layers at P12 (Figure S3B). At two months of age, no retina tissue was left in the mutant retina (Figure S3C).

### Loss of *Tmem30a* in cone cells leads to cone-photoreceptor defects

**Figure 2:**
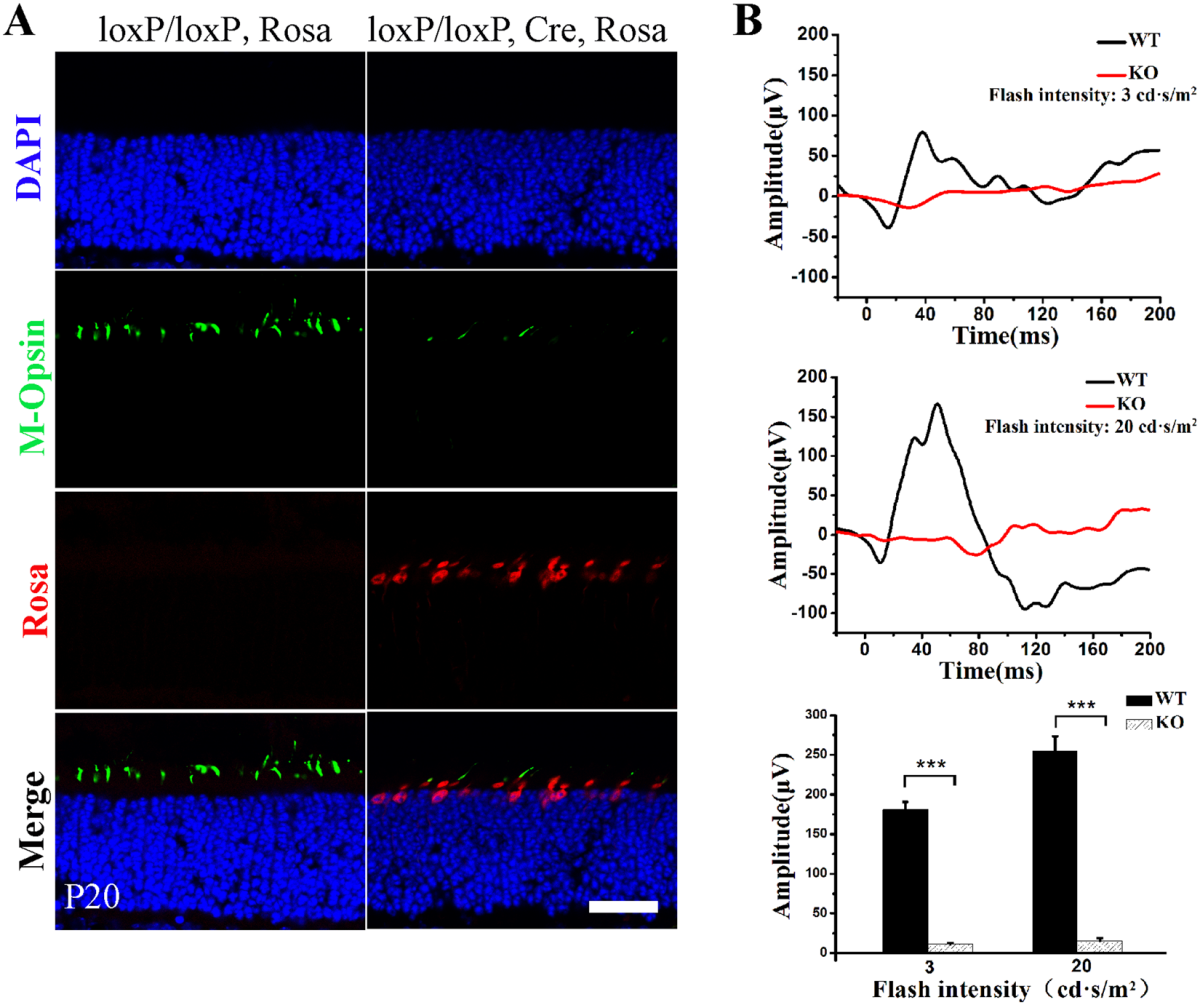
The deletion of *Tmem30a* in cone cells leads to cone cell degeneration. (**A**) The generation of cone-specific *Tmem30a*-knockout mice using HRGP-Cre transgenic mice. A ROSA-TdTomato reporter was introduced into the mice to monitor Cre activity. Mice with the genotype HRGP-Cre or *Tmem30a* ^*loxp*/*loxp*^ were designated as controls. Loxp/loxp Cre + Rosa mice were designated with the genotype *Tmem30a* ^*loxp*/*loxp*^ HRGP-Cre, ROSA-TdTomato. TdTomato is a red fluorescent protein variant that was expressed in the presence of HRGP-Cre. Therefore, Cre-positive cells were also deficient in *Tmem30a*. Compared with controls, Green/Red opsin expression (green) was lost in Cre-expressing cone cells (red, Tomato-positive cells). Sections were counter stained with DAPI (blue). Scale bar is 25 μm. (**B** and **C**) Functional characteristics of *Tmem30a* cone knockout mice. Representative ERG traces of the retinas in 1-month-old *Tmem30a* cone knockout mice under photopic conditions. (**D**) Statistical analysis of the amplitudes of b wave in the mice under photopic condition. Values in C represent the means +/− SEMs of four mice. N = 5. ***P < 0.001. Scale bar: 25 μm.

To investigate the role of *Tmem30a* in photoreceptor cells, we generated a cone-photoreceptor knockout line using human red/green pigment gene promoter (HRGP-Cre) mice^36^. In the HRGP-Cre mouse, Cre expression is initiated on postnatal day 7–10 and is specific for M-opsin-expressing cone cells^20^. Mice homozygous for the *Tmem30a* floxed allele (*Tmem30a* ^*loxp*/*loxp*^) were mated to HRGP-Cre (cone-Cre), *Tmem30a* ^*loxp*/+^ mice to generate *Tmem30a* ^*loxp*/*loxp*^ HRGP-Cre (mutant) and *Tmem30a* ^*loxp*/+^ HRGP-Cre (control) mice. Cre-positive controls were used in the analysis to ensure that any phenotypic difference between mutants and controls was due to the loss of *Tmem30a*. The *rd1* mutation in the original HRGP-Cre mice was screened out by mating them to C57BL/6J mice before the experiment. We verified the specificity of HGRP-Cre using a TdTomato reporter. To do this, we crossed ROSA26-TdTomato reporter mice to *Tmem30a* ^*loxp*/*loxp*^ HRGP-Cre mice to generate *Tmem30a* ^*loxp*/*loxp*^ HRGP-Cre Rosa-TdTomato mice. In the presence of the Cre enzyme, the stop codon before the TdTomato expression cassette was removed, and Rosa was expressed in Cre-positive cells (Fig. 2A). In Cre-expressing cells, M-opsin expression was greatly diminished (Fig. 2A), suggesting that *Tmem30a* function is important for M-opsin expression and stability. Photopic ERG analysis revealed diminished ERG responses in the cone mutant retina (Fig. 2B,C). No recognized a-wave or b-wave was observed in cone-knockout mice.

**Figure 3:**
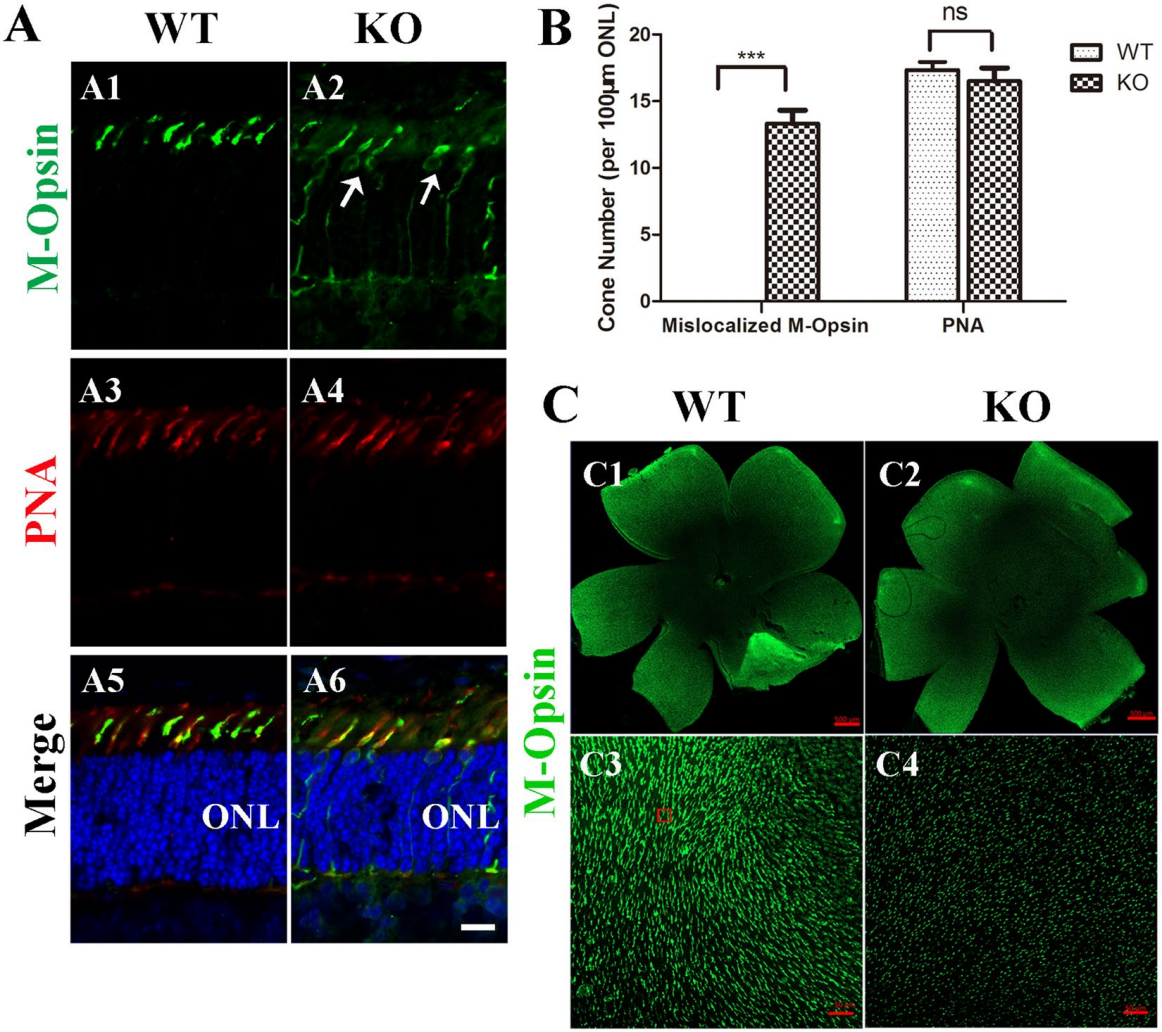
Cone opsin trafficking is impaired in *Tmem30a* cone mutants. (**A**) Retina cryosections from P16 control and mutant littermates were immunostained with M opsin (green), PNA (red) and DAPI (blue). In the control retina, M opsin localized to the outer segment of cone cells. However, in mutant cone cells, M opsin accumulated in the inner segment and in cell bodies (arrows). (**B**) Quantification of cone cells with mislocalized M opsin. Cells were counted on 100μm ONL segment at 500 μm to the optic nerve. N = 6. ***P < 0.001. (**C**) Retina whole mounts from control (WT) and *Tmem30a* cone mutant (TmCone KO) littermate mice were immunostained with an antibody to label M opsin. Scale bar is 500 μm. In the wild-type retina, M cone cells were evenly spaced (C3). In the mutant retina, the number of M cone cells was similar. However, the cone outer segment was shorter (C4). Scale bar: 25 μm.

Close examination of P16 cross-section retinas in higher-magnification images revealed mislocalization of M-opsin to the inner segment (IS) and to the cell bodies, while there was no obvious loss of cone cells (arrows, Fig. 3A,B). No protein expression can be observed for GRK1 and CNG3, two proteins expressed in cones (Figs S6 and 7). No mislocalized GRK1 or CNG3 was observed in the cell body, probably due to rapid degradation of these proteins in mutant cones. After P16, the number of cone cells in the mutants declined quickly, such that very little cone opsin staining could be seen in the cone outer segment at P42 (Figure S4A). We next analysed the number of cone cells in the mutant mice by immunostaining whole-mount retinas with M-opsin. This whole-mount study enabled us to survey cones across the whole retina and to avoid possible artefacts introduced by examining sections from different places in the retina. At P16, approximately equal numbers of cone outer segments were found in the mutant and the control mice (Fig. 3C). At P42, no cone cells were observed (Figure S4B4). In the P42 *vmd* mutant retina, there were similar numbers of cone cells to those in controls (Figure S5). However, close examination of the cross-section of retinas in higher-magnification images revealed a reduced amount of opsin in the cone OS and showed that the OS did not develop properly (Figure S5). Apparently, loss of *Tmem30a* in cone cells leads to a more severe phenotype than the loss of *Atp8a2* in *vmd* mice (Figs S4 and 5). Because TMEM30A binds to multiple PS flippases^20, 24^, including ATP8A2, these data suggest that other PS flippase(s) may play a role in the retina.

### Deletion of *Tmem30a* in adult animals leads to rod cell degeneration

**Figure 4:**
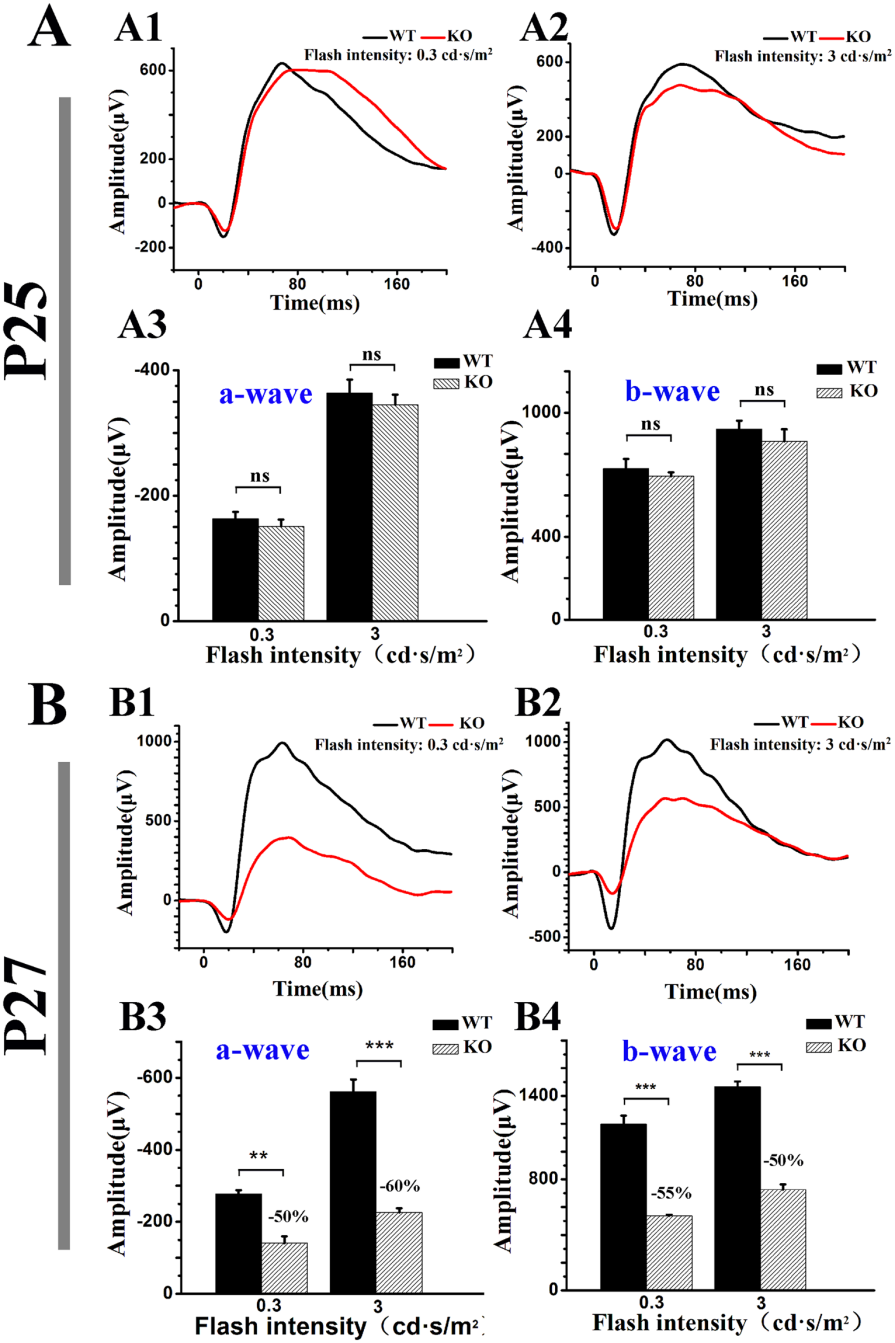
Functional analysis of *Tmem30a* inducible knockout mice. Representative ERG traces of the retinas in *Tmem30a* inducible knockout mice under scotopic conditions at P25 (A1–2) and P27 (B1–2). Statistical analysis of the amplitudes of a, b waves in the mice under scotopic condition were listed in A3–4, B3–4. No significant difference was observed at P25 on scotopic ERG a wave and b wave amplitudes. However, the amplitudes of both a wave and b wave in mutant retina were lower that that of controls (B1–4). Values in B represent the means +/− SEMs of four mice. ***P < 0.001.

To confirm our finding with M-opsin-Cre and to assess the role of *Tmem30a* in developed photoreceptor cells, we used tamoxifen-activated CAG-CreER^37^ to remove *Tmem30a* in adult mice. In CAG-CreER animals, Cre is broadly expressed under the control of a chicken beta actin promoter coupled with a cytomegalovirus (CMV) immediate-early enhancer promoter. The Cre recombinase is fused to a mutant form of the mouse oestrogen receptor ligand-binding domain, which does not bind natural ligand at physiological concentrations but will bind the synthetic ligand, 4-hydroxytamoxifen. Upon induction with tamoxifen, CAG-CreER induces the deletion of loxP-flanked exon 3 in all cells of the retina. We treated P20 animals with tamoxifen and assessed the retina at P25, P27 and P30. Upon induction, *Tmem30a* was efficiently deleted in the retina. We utilized ERG analysis to assess physiological status of the retina. At P25, no visible difference was observed in the mutant retina (Fig. 4A). However, at P27, ERG test revealed reduced amplitudes for both a waves and b waves (Fig. 4B). The amplitude of a wave in the mutant retina was reduced to 50% of that of controls (Fig. 4B3). The amplitude of b wave in the mutant retina was reduced to 40% of that of controls (Fig. 4B4).

**Figure 5:**
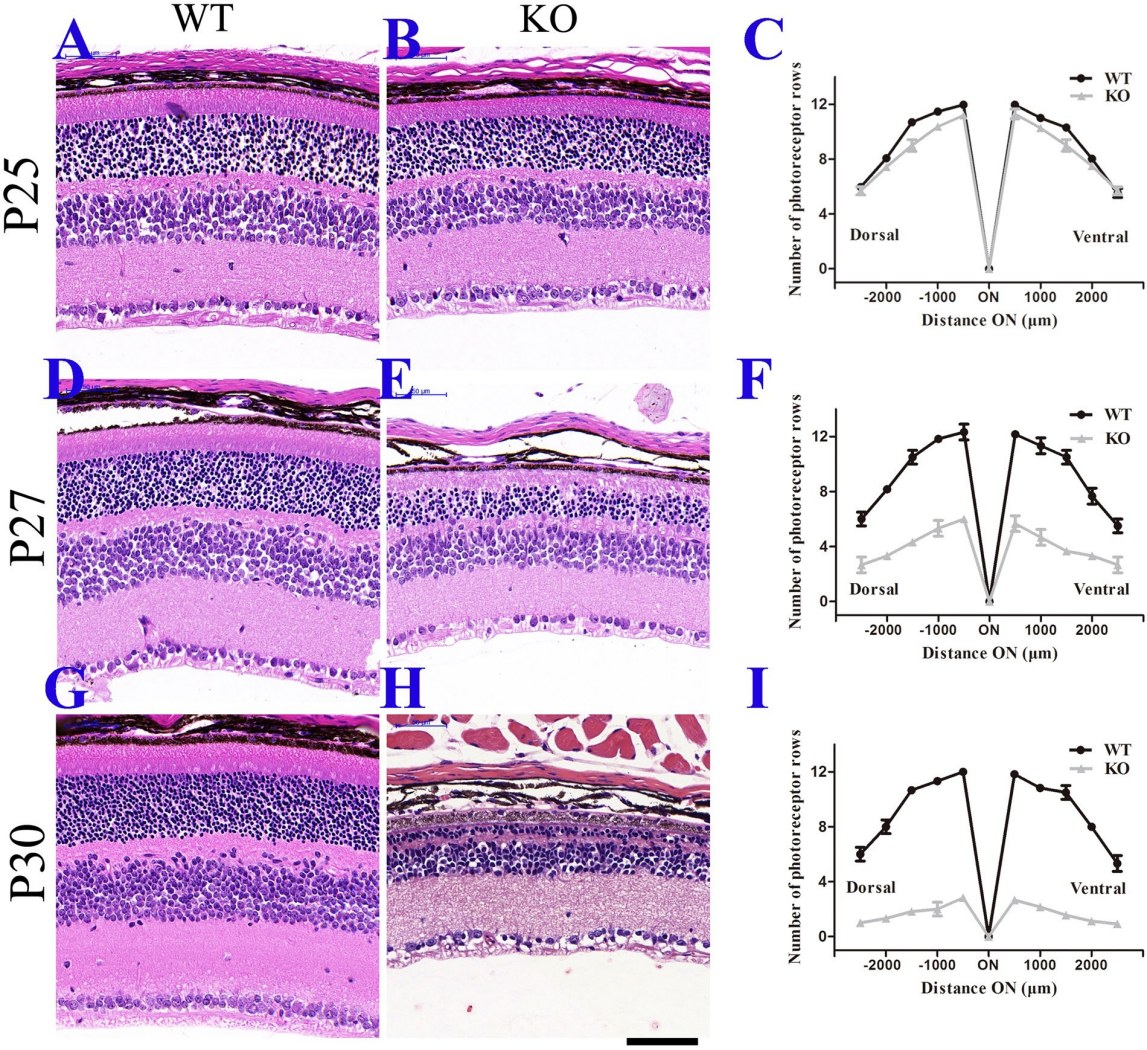
Retinal degeneration in *Tmem30a* inducible knockout mutants. Paraffin sections of retinas from P25 (**A**,**B**,**C**. 5 days after induction), P27 (**D**,**E**,**F**. 7 days after induction) and P30 (**G**,**H**,**I**, 10 days after induction) were stained with H&E. Quantification of outer nuclear layer (ONL) nuclei revealed that the mutant ONL was reduced to approximately 50% of the control (**F**) at P27. At P30, the ONL was reduced to 2–3 cells per row in the mutant retina, compared with 10–11 cells per row in the control retina (**I**). Nuclei were counted every 200 500 μm to the optic nerve. N = 6, ***P < 0.001. Scale bar: 25μm.

**Figure 6:**
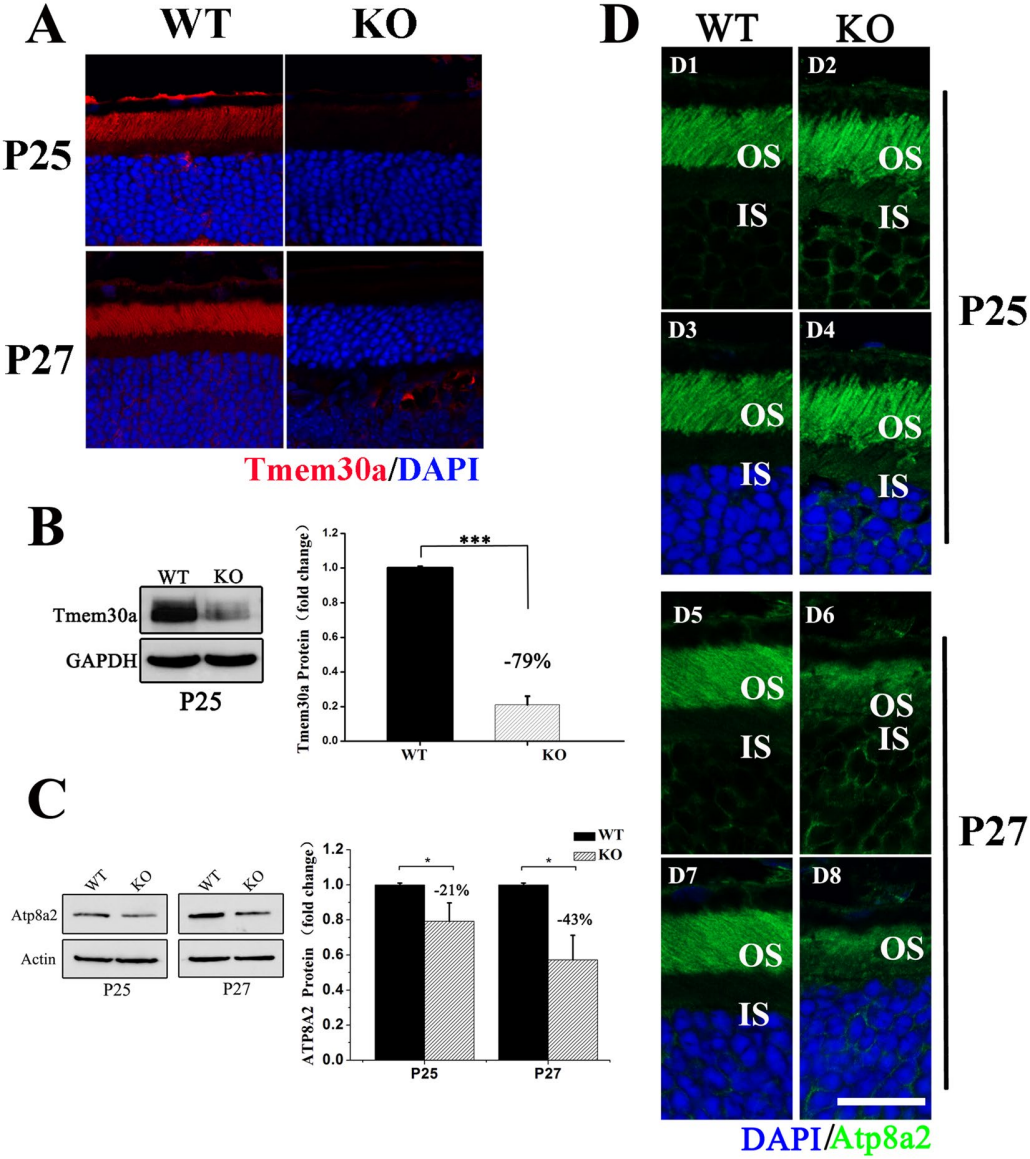
Analysis of TMEM30A and ATP8A2 expression in the retina of *Tmem30a* inducible knockout mutant mice by immunofluorescence microscopy. (**A**) Immunofluorescence labeling of retina cryosections from control (WT) and mutant (KO) littermates at P25 (5 days after induction) and P27 (7 days after induction) using TMEM30A antibody (red) and DAPI (blue). (**B**) Western blot analysis of TMEM30A in wild-type (WT) and knockout mice using TMEM30A antibody. GAPDH was used as a loading control. Quantification of TMEM30A revealed that expression of TMEM30A was decreased to 21% of that of control. Gel picture was cropped to save space. Full gel picture was listed as Figure S11. (**C**) Western blot analysis of ATP8A2 in wild-type (WT) and knockout mice using ATP8A2 antibody. Actin was used as a loading control. Quantification of ATP8A2 in western blotting data in (**C**) revealed that at P25, expression of ATP8A2 was decreased to 79% of that of control. At P27 (7 days after induction), ATP8A2 expression was further reduced to 57% of that of control. Gel picture was cropped to save space. Full gel picture was listed as Figure S12. ***P<0.001﻿. (**D**) Immunofluorescence labeling of retina cryosections in control (WT) and mutant (KO) littermates at P25 (5 days after induction) and P27 (7 days after induction) using ATP8A2 antibody (green) and DAPI (blue). ATP8A2 mislocalized to the inner segment and cell bodies of retina in the mutant (KO). Scale bar: 25 μm.

We examined tissue sections stained with haematoxylin and eosin (H&E) to assess the structures of mutant retinas at P25, P27 and P30. At P25 no obvious difference was observed between the mutant and the control mice (Fig. 5A,B,C). However, at P27, a shorter outer segment and a thinner outer nuclear layer (ONL) were seen in the mutant retina (Fig. 5D,E,F). The mutant ONL was reduced to approximately 50% of the control (Fig. 5F). There were also few cells in the retina ganglion cell layer. At P30, no OS was left, and the ONL was reduced to 2–3 cells per row in the mutant retina, compared with 10–11 cells per row in the control retina (Fig. 5G,H,I). To investigate the molecular mechanisms underlying these severe phenotypes, we examined retina sections by immunohistochemistry using various antibodies. At P25 and P27, no TMEM30A staining wad observed in the photoreceptor layer in mutant retina, confirming loss of TMEM30A (Fig. 6A). At P25, the expression level of TMEM30A in mutant retina was reduced to 21% of that of controls (Fig. 6B). ATP8A2, the P4-ATPase expressed in the retina, requires its beta subunit TMEM30A for subcellular localization. To assess the effect of *Tmem30a* deficiency on ATP8A2 in the retina, we examined expression and localization of ATP8A2 in *Tmem30a* mutant retina. At P25, the expression level of ATP8A2 was reduced to 79% of that of controls, while at P27 ATP8A2 expression level in the mutant retina was reduced to 57% of that of controls (Fig. 6C). Immunostaning revealed that ATP8A2 was mislocalized to the inner segment and cell bodies in the mutant retina (Fig. 6D).

**Figure 7:**
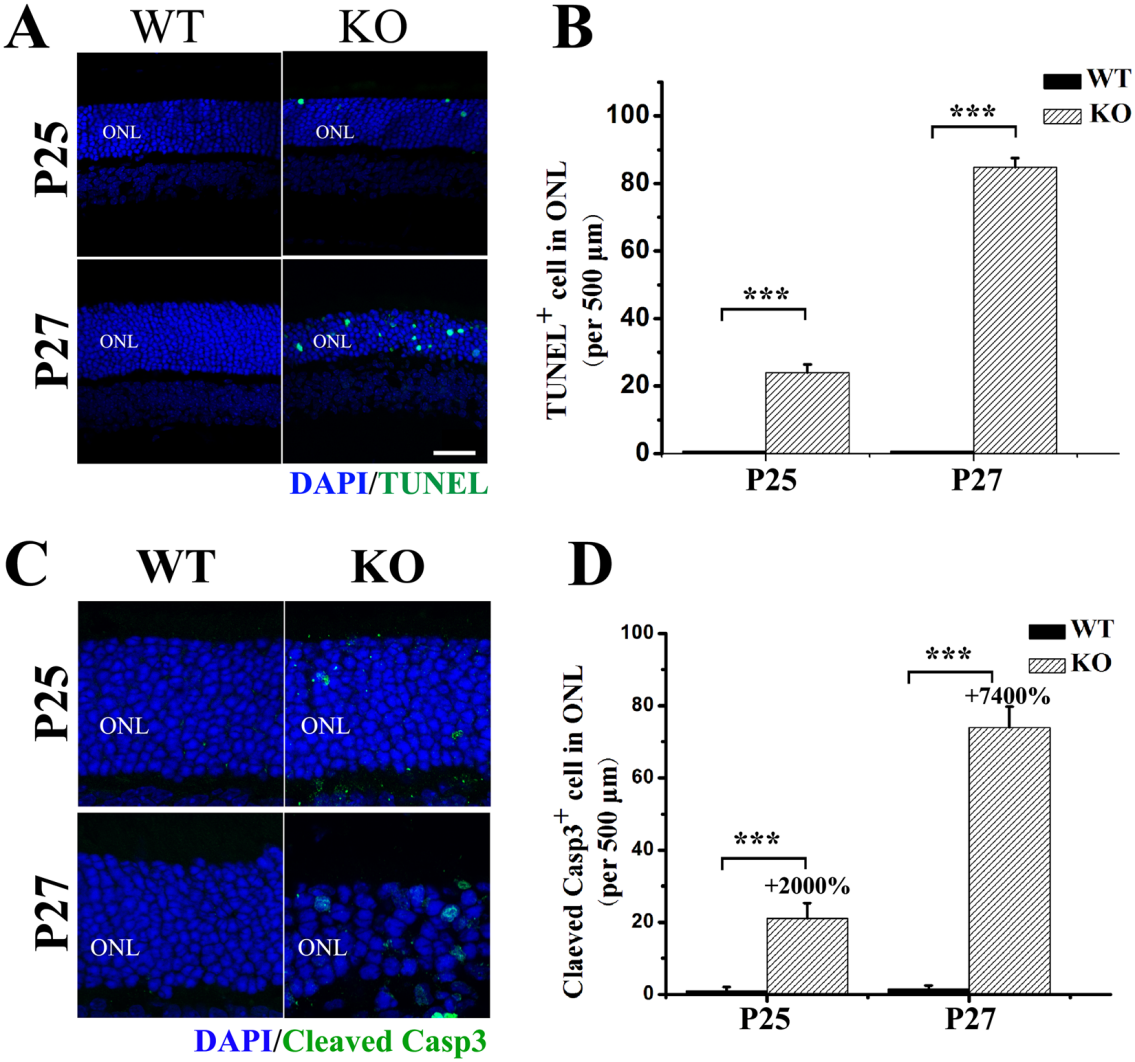
Increased apoptosis in *Tmem30a* inducible knockout mutants. (**A**) Immunofluorescence labeling of retina cryosections from P25 (5 days after induction) and P27 (7 days after induction) control and mutant littermates with the TUNEL labeling kit. TUNEL staining is shown in green and DAPI in blue. There were no TUNEL positive cells in the control sections. At P25, 22 TUNEL positive cells were observed in 500 μm segment in mutant mice. At P27, 82 TUNEL positive cells were observed in 500 μm segment in mutant mice. N = 6. (**C**,**D**) Immunofluorescence labeling of retina cryosections from P25 and P27 control and mutant littermates with cleaved caspase 3 (green) and DAPI (blue). Caspase 3 staining positive cells increased dramatically at both P25 and P27 in mutant mice. N = 6. P<0.001. Scale bar: 25μm.

PS exposure on the cell surface acts as an “eat-me signal” to mark apoptotic cells for macrophage clearance^22, 38^. The loss of PS asymmetry in the *Tmem30a*-mutant retina prompted us to examine the apoptotic signalling pathway in the mutant retina. At P25, compared with the control retina, TUNEL staining increased in the mutant retina (Fig. 7A,B), and at P27, more positive staining cells were observed (Fig. 7A,B). Cleaved caspase 3, an important molecule for cell apoptosis, was up-regulated in P25 and P27 retina (Fig. 7C,D). These data indicated that the loss of TMEM30A activity in the retina led to the initiation of the cell apoptosis pathway, which explains the rapid loss of photoreceptor cells in mutant retina.

### Loss of PS flippase activity and PS asymmetry in mutant MEF cells

**Figure 8:**
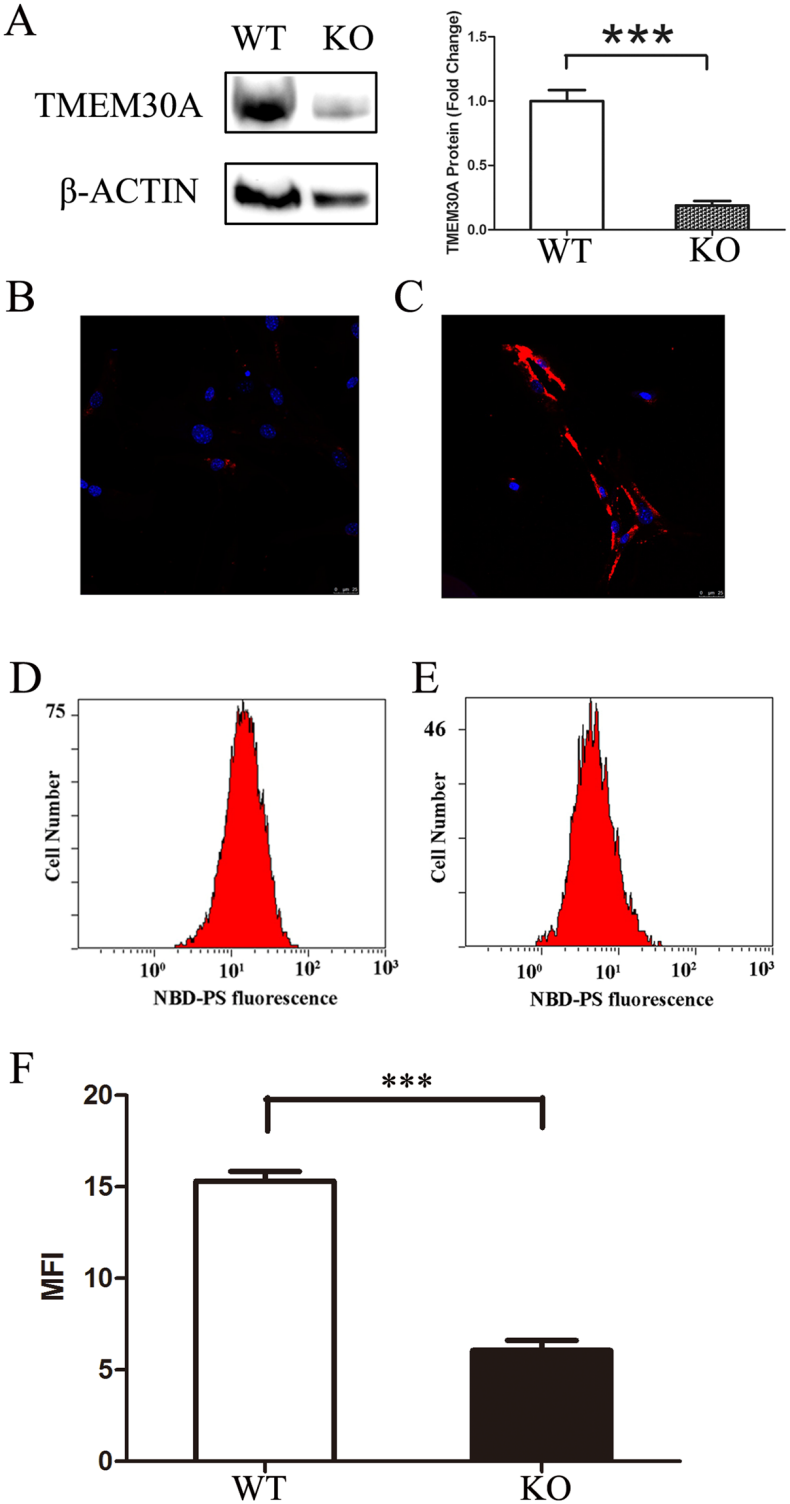
Loss of PS flippase activity in *Tmem30a*-mutant MEFs. (**A**) Western blot analysis revealed diminished *Tmem30a* expression in mutant MEFs. The expression of TMEM30A in mutant MEFs was reduced to 30% of that of control cells. Gel picture was cropped to save space. Full gel picture was listed as Figure S13. P<0.001. (**B**,**C**) Annexin V staining showed PS exposure on the mutant MEF cell surface, not on control cells. (**D**–**F**) NPD-PS labelling assay revealed loss of PS flippase activity in mutant MEFs. Internalization of NBD phospholipids by control (**D**) and mutant MEF cells (**E**). Loss of *Tmem30a* led to decreased NBD-PS uptake (**E**). The Y-axis showed numbers of NBD-PS-labelled MEF cells. The X-axis represented NBD-PS fluorescence intensity of intact living cells. Mutant cells exhibited decreased fluorescence intensity, compared to control cells (**F**). MFI, median fluorescence intensity. N = 3. ***P<0.001.

To investigate the cellular pathways in *Tmem30a* knockout cells, we generated MEFs using *Tmem30a* ^*loxp*/*loxp*^ CAG-CreER and *Tmem30a* ^*loxp*/*loxp*^ embryos. The addition of tamoxifen to the culture medium could effectively remove the loxp-flanked exon 3, generating mutant MEFs. TMEM30A expression in mutant MEFs was reduced to 20% of control cells (Fig. 8A). Mutant MEFs grew normally, and no apparent morphological abnormalities could be observed. However, annexin V staining revealed a loss of PS asymmetry and more exposure of PS on the outer membrane in mutant MEFs (Fig. 8B,C). In agreement with this finding, loss of *Tmem30a* led to decreased fluorescence NBD-PS uptake. PS flippase activity in mutant cells was reduced to 30% of control cells (Fig. 8D–F). Compared to wildtype cells, the expression levels of *Atp8a2*, *Atp8b4* and *Atp11a*, *Atp11c* were reduced in mutant MEFs (Figure S8). These data demonstrated that loss of *Tmem30a* resulted in reduced PS flippase activity in the cell.

## Discussion

In this study, we analysed the role of *Tmem30a* in the retina by generating novel retina-specific knockout mice. *Tmem30a*-knockout mice were embryonic lethal, revealing the importance of this gene *in vivo* (Table S1). The pan-neuronal knockout of *Tmem30a* using Nestin-Cre also led to embryonic lethality (Table S2). Therefore, *Tmem30a*-knockout mice exhibit more severe phenotypes than the five available PS flippase-knockout mice, including *Atp8b3*, *Atp8b1*, *Atp8a2*, *Atp8a1* and *Atp11c*. This finding is in agreement with previous reports showing that TMEM30A binds to multiple P4-ATPases^23, 26^.

*Tmem30a* deficiency in the cone cells led to diminished photopic ERG responses and the rapid degeneration of cone cells (Figs 2–3, S4). At P16, opsin accumulated in the cone cell bodies (Fig. 3A) and lead to the death of affected cells. At P16, there were similar numbers of M-opsin-positive cells in the mutant retina and the controls (Fig. 2C). However, the cone OS appeared shorter, and by P42, no cone cells were left (Figure S4). By contrast, in *vmd* mice, no apparent accumulation of opsin in the IS and cell body could be seen at P42, although the opsin-positive OS was shorter (Figure S5). Apparently, the loss of *Tmem30a* in cone cells resulted in more severe phenotypes than the loss of *Atp8a2*, which is likely due to the fact that TMEM30A binds multiple P4-ATPases in the retina.

In *Tmem30a*-mutant rod cells, ATP8A2 expression was reduced and ATP8A2 mislocalized to the IS and cell bodies (Fig. 6C,D), and these data are consistent with previous reports that used a *Tmem30a*-knockout cell line. Segawa *et al*. showed that the loss of ATP11C in the human KBM7 cell line resulted in reduced PS flippase activity^22^. Moreover, the internalization of NBD-PS was completely defective in *Tmem30a*-mutant cells, and *Tmem30a*-mutant cells exposed PS on the cell surface^22^. Indeed, in *Tmem30a*-deficient MEFs, the loss of *Tmem30a* leads to decreases in PS flippase activity and exposure of PS on the outer leaflet, as revealed by annexin V staining (Fig. 8). In the retina, the loss of *Tmem30a* resulted in rod cell apoptosis (Fig. 7). The rapid degeneration of rod cells was likely to be mediated through the apoptotic pathway.

Mutations in *Atp8a2* in mice have been shown to cause axonal degeneration and the degeneration of both rod and cone cells in the retina^16^. In addition, disruption of *ATP8A2* leads to severe neurological disorders that are characterized by cerebellar ataxia, mental retardation and disequilibrium syndrome in humans. A missense mutation (NM_016529 c.1128C > G, p.I376M) was identified in three patients from a consanguineous family in Turkey^15^, and these patients exhibited encephalopathy, cerebellar atrophy, body tremor and mental retardation. These patients were able to sit with no support and crawl on their hands and feet, although no ophthalmology evaluation was performed on these patients due to the lack of consent. Very recently, two unrelated patients were reported to exhibit encephalopathy, mental retardation, severe hypotonia, a normal retina and optic atrophy^14^. The mutations that were uncovered included a homozygous mutation of *ATP8A2*, c.1287G > T, p.K429N, and the compound heterozygous mutations c.1630G > C, p.A544P and c.1873C > T, p.R625W, emphasizing the importance of PS flippases in the central nervous system^14^. Based on our mouse data, homozygous mutations that affect the function of *Tmem30a* are likely to be embryonic lethal. Given the finding that *Tmem30a* is broadly expressed in various tissues (Figure S1) and binds to multiple P4-ATPases, its roles in different tissues warrant further investigation.

Collectively, our data demonstrate novel roles for *Tmem30a* in retinal photoreceptor cells. *Tmem30a* deficiency resulted in a diminished photopic ERG response and in the accumulation of opsin in the inner segment and in cell bodies in cone cells. The deletion of *Tmem30a* also resulted in a loss of PS flippase activity and the exposure of PS on the cell surface in MEFs. Together, our studies highlight the importance of *Tmem30a* in the retina.

## Methods

### Experimental animals and genotyping

All animal study protocols were approved by the Animal Care and Use Committee of the Hospital of the University of Electronic Science and Technology and Sichuan Provincial People’s Hospital. All experimental procedures and methods were carried out in accordance with the approved study protocols and relevant regulations. Mice were raised in cyclic lighting conditions with a 12-hour light and 12-hour dark cycle.

The *Tmem30a*-knockout first targeting vector was obtained from the Knockout Mouse Project (KOMP) Repository. The targeting vector was linearized by AscI and transfected by electroporation of 129/SvEv embryonic stem cells. Homologously recombined positive clones were selected by neomycin, screened by PCR and confirmed by long-range PCR at both the 5′ and the 3′ arms. The primers used for long-range PCR for the 5′ arm were as follows: Tmem30a-GF3, GAGGAAGCGGAAGTGTAAGTTACCAAG; and Tmem30a-LAR3, CACAACGGGTTCTTCTGTTAGTCC. The primers used for long-range PCR for the 3′ arm were as follows: Tmem30a-GR3, GTGTGAAGTCAACGTCATTATCGGAGAATC; and Tmem30a-RAF5, CACACCTCCCCCTGAACCTGAAAC. Two independent clones of the targeted stem cells were microinjected into C57BL/6 J blastocysts. Resulting chimaera male mice were mated to C57BL/6 J female mice to generate F1 heterozygous founders. Heterozygous F1 (designated as *Tmem30a* ^*tm1Xjz*^) mice were crossed to generate F2 offspring. To generate a conditional allele of *Tmem30a*, heterozygous F1 mice were crossed to Flper mice^33^ (strain name: B6.129S4-Gt(ROSA)26Sortm1(FLP1)Dym/RainJ, stock number 009086, also named ROSA26::FLPe knock in, https://www.jax.org/strain/009086) to remove the FRT-flanked LacZ reporter and neomycin cassette. The resulting conditional allele of *Tmem30a* was designated as *Tmem30a* ^*tm1.1Xjz*^. *Tmem30a* ^*tm1.1Xjz*^ mice (for simplicity, designated as *Tmem30a* ^*loxp*^) were backcrossed to C57BL/6 J mice for 5 generations.

*Tmem30a* ^*loxp*/+^ mice were mated to Nestin-Cre^34^ mice to generate *Tmem30a* ^*loxp*/+^ Nestin-Cre mice. *Tmem30a* ^*loxp*/+^ Nestin-Cre mice were crossed to *Tmem30a* ^*loxp*/*loxp*^ homozygous mice to generate a pan-neuronal deletion of *Tmem30a*. *Tmem30a* ^loxp^ Six3-Cre mice were generated by crossing *Tmem30a* ^*loxp*/+^ mice to the Six3-Cre line^35^. Cone-specific knockout mice were generated by crossing *Tmem30a* ^*loxp*/+^ to HRGP-Cre mice^36^. The *rd1* mutation was bred out before the experiment. *Tmem30a* ^*flox*/+^mice were crossed to the broadly expressed inducible CRE CAG-Cre-ER mice^37^ (stock Tg(CAG-cre/Esr1*) 5Amc/J, https://www.jax.org/strain/004453) to generate inducible adult knockout mice. To monitor the efficiency of Cre-mediated deletion of the floxed exon, a TdTomato reporter was used (strain name: B6.Cg-Gt(ROSA)26Sortm14(CAG-TdTomato)Hze/J, also named Ai14D, http://jaxmice.jax.org/strain/007914.html)^38^. The reporter contains a loxP-flanked STOP cassette that prevents transcription of the downstream CAG promoter-driven red fluorescent protein variant TdTomato. In the presence of Cre recombinase, the reporter mice will have the STOP cassette removed in the Cre-expressing tissue(s) and will express TdTomato. Because this CAG promoter-driven reporter construct was inserted into the Gt(ROSA)26Sor locus, TdTomato expression was determined by the tissue(s) that expressed Cre recombinase.

The Atp8a2 mutant *vmd* strain was purchased from The Jackson Laboratory and was described in Zhu *et al*.^16^. *vmd* is a 9167 bp genomic deletion that results in removal of the entire exon 32 of *Atp8a2*. This results in a 32 amino acid deletion in the ninth transmembrane domain of ATP8A2 and disrupted the function of ATP8A2^16^. This allele was backcrossed to C57BL/6 J for 10 generation to remove *rd1* mutation in the original C3H background and generated congenic strain of *vmd* on C57BL/6 J. As standard practice for *vmd-*mutant mice, dry food was supplemented with a soft maintenance diet (DietGel 76 A, Clear H_2_O, Portland, ME, USA).

### Tamoxifen treatment

A total of 100 mg of tamoxifen salt (Sigma, St Louis, MO, USA) was dissolved in 10 ml of ethanol as a stock solution. On the day of injection, a 1 mg/ml working solution was prepared by mixing 10 mg/ml stock solution with corn oil (Sigma, Sigma, St Louis, MO, USA) and mixed well. On P20, mutant and control mice were intraperitoneally injected with a daily dosage of 25 mg/Kg body weight for three days.

### Genotyping by PCR

Genomic DNA extracted from mouse-tails was amplified by PCR using primers for the *Tmem30a* gene (Tmem30a-Loxp2-F, 5′-attccccttcaagatagctac -3 and Tmem30a-**Loxp**2-R, 5-aatgatcaactgtaattcccc -3). Amplification was performed using a master mix (Invitrogen, USA). The first cycle consisted of 95 °C for 2 minutes, followed by 33 cycles of 94 °C for 15 seconds, 58 °C for 20 seconds and 72 °C for 30 seconds. Cre was genotyped using generic Cre primers: Cre-F, TGCCACGACCAAGTGACAGCAATG, and Cre-R, ACCAGAGACGCAAATCCATCGCTC). TdTomato mice were genotyped using the following protocol provided by the JAX mouse service: oIMR9020, 5′-AAGGGAGCTGCA GTGGAGTA-3′; oIMR9021, 5′-CCGAAAATCTGTGGGAAGTC-3′; oIMR9103, 5′--GGCATTAAAGCAGCGTATCC-3′; and oIMR9105, 5′-CTGTTCCTGTACGGCATGG-3′. FLPer mice were genotyped using the following primers: oIMR1348, 5′-CAC TGATATTGTAAGTAGTTT gC-3′; oIMR1349, 5′-CTA GTGCGAAGTAGT GATCAGG-3′; oIMR1427, 5′-TGTTTTGGAGGCAGGAAGCACTTG-3′; and oIMR1428, 5′-AAATACTCCGAGCGGATCACAAG-3′.

For the *vmd* genomic deletion, three primers flanking the region were used to distinguish between the wild-type and mutant alleles: vmdF, 5′-CTAACTGTGGCTCACTTACCTCCT-3′; vmdR1, 5′-TCCTCCAGAACATTGAAGTGACTA-3′; and vmdR2, 5′-TGCATCTTGATTTTTGCTTTGTAT-3′. A 403-bp amplicon was produced in the presence of the wild-type allele using the vmdF and vmdR1 primers, and a 207-bp amplicon was produced in the presence of the mutant *vmd* allele using the vmdF and vmdR2 primers.

### RT-PCR

Tissues from 1- to 2-month-old animals were dissected and placed into RNAlater (Ambion, Austin, TX, USA) at room temperature. Total RNA was prepared from these tissues using TRIzol reagent (Life Technologies) according to the manufacturer’s instructions. RNA samples were treated with RNase-free DNaseI (Ambion, Austin, TX, USA) to remove genomic DNA, and the RNA concentration was determined with a NanoDrop (ND-1000) spectrophotometer. A total of 3 µg of RNA was reverse transcribed using random primers and the MessageSensor RT kit (Ambion, TX, USA). The primers used for RT-PCR for mouse gene studies were listed in the material and method section of supplementary data. PCR was performed with Taq polymerase (New England Biolabs, MA, USA), and the PCR products were resolved on 3% agarose gels.

### Histology and measurement of outer nuclear layer

For haematoxylin and eosin staining (H&E), eyes from WT and knockout (KO) mice were removed, marked on the nasal side for orientation and fixed overnight in 1.22% glutaraldehyde and 0.8% paraformaldehyde in 0.08 M phosphate buffer, embedded in paraffin, and cut into 5-µm sections. H&E stained sections were used to count the rows of photoreceptors in the outer nuclear layer^36^. Three measurement of the outer nuclear layer were taken every 200 μm from the optic nerve and averaged. The optic nerve was designated as 0 µm.

### Electroretinograms

Control and mutant mice were dark-adapted overnight, and all subsequent procedures were performed under dim red light. Animals were anesthetized with a combination of ketamine (16 mg/kg body weight) and xylazine (80 mg/kg body weight) in normal saline. Dark-adapted ERGs were recorded on with the responses to short-wavelength flashes over 4.0 log units to the maximum intensity with the Espion Visual Electrophysiology System from Diagnosis, LLC (Littleton, MA, USA). Cone-mediated ERGs were recorded with white flashes after 20 min of complete light adaptation.

### Immunohistochemistry

For immunohistochemistry, enucleated eyes were removed, marked at the nasal side for orientation and fixed for 1 hour in 4% paraformaldehyde in 100 mM phosphate buffer (PB) (pH 7.4) and then cryoprotected in 30% sucrose. Tissues were embedded in optimal cutting temperature solution (OCT) and frozen on dry ice for sectioning. Sections were blocked and permeabilized with 10% normal goat serum and 0.2% Triton X-100 in phosphate buffer for 30 minutes. Labelling with various antibodies was performed as previously described^16^. Mouse monoclonal against cleaved caspase 3 was purchased from R&D Systems (Minneapolis, MN, USA). Primary antibodies were diluted in phosphate buffer containing 5% normal goat serum and 0.1% Triton X-100 at the following concentrations: ATP8A2 polyclonal antibody (purified 0.3 µg/ml), TMEM30A monoclonal antibody (Cdc50-7F4), PMC 1D1 monoclonal antibody to the CNGA1 channel subunit, Rho1D4 monoclonal antibody to rhodopsin were gifts from Dr. Robert Molday, University of British Columbia, Canada. GRK1 antibody, Alex-Fluor-594-labelled annexin V (Invitrogen, Shanghai, China). Sections were incubated with primary antibodies overnight. Then, the sections were washed with PB three times and labelled for 1 hour with Alexa-Fluor-488- or Alexa-Fluor-594-labelled goat anti-mouse or anti-rabbit Ig secondary antibody (diluted 1:500) and counterstained with DAPI.

Quantification of mislocalized M-Opsin in the cell bodies of cones was performed as following: P16 cross-section retinas were staining with M-Opsin opsin (AB5405, AB5407; Millipore, MA, USA) and DAPI and higher-magnification images were captured on a Leica SP8 confocal microscope. The number of cones with mislocalized M-Opsin to the inner segment (IS) and to the cell bodies was counted on 100 µm ONL segment at the dorsal side 500 µm to the optic nerve.

Retinal whole mounts were prepared as previously described^16^. Mouse eyes were lightly fixed for 1 hour after light onset with 4% paraformaldehyde for 15 minutes. The retina was dissected from the retinal pigment epithelial layer and fixed for another 15 minutes. After washing in 10 mM HEPES, pH 7.4, 140 mM NaCl and 2.5 mM CaCl_2_, the retinas were labelled with M-Opsin antibody for 24 hours in 10 mM HEPES, pH 7.4, 140 mM NaCl, and 2.5 mM CaCl_2_. The retinas were then washed and relabelled with the Alex-Fluor-594 (Molecular Probes, Eugene, OR, USA) secondary antibody. After labelling with the secondary antibody, the retinas were counter stained with DAPI, mounted on slides with the photoreceptor outer segments facing up, and photographed on a Zeiss LSM 800 confocal scanning microscope. For annexin V staining, Alex-Fluor-594-labelled annexin V (Molecular Probes, Eugene, OR, USA) was incubated with the retinas for 3 hours, and sections were counterstained with DAPI.

### Cell culture

HEK-293T and COS7 cells (American Type Culture Collection (ATCC, Manassas, VA, USA) were seeded on coverslips and transiently cultured in DMEM medium with high glucose (HyClone) supplemented with 10% foetal bovine serum and 1% (vol/vol) penicillin/streptomycin at 37 °C in a 5% CO2 atmosphere. Cells were seeded in six-well plates (Corning, NY, USA) and transfected at 50% confluency with 1 µg of pCMV6-AN-Flag-*Atp8a2, pCMV6-Tmem30a-*HA or empty vectors using Lipofectamine 3000 (Invitrogen) according to the manufacturer’s instructions. Cells were harvested after 24–48 hours and fixed in 4% paraformaldehyde for 15 min at room temperature. After blocking with 1 × PBS containing 5% normal goat serum and 0.2% Triton X-100, cells were incubated with anti-Flag antibody (Sigma, St. Louis, MO, USA) and anti-HA antibody (Roche, Redwood city, CA, USA) at 4 °C overnight. Alexa-Fluor 594/488-conjugated secondary antibody (Invitrogen) was applied, and nuclei were counter stained with DAPI. Images were acquired with a confocal system (Leica) using a 63 × 1.4 NA oil immersion objective lens.

### Mouse embryonic fibroblast (MEF) isolation

MEFs were isolated according to a previously reported protocol^39^.

### Lipid translocation assay

Lipid translocation assay was performed following previous published methods^16, 22^.

The labeled phospholipid analog 16:0–06:0 NBD-PS [1-palmitoyl-2-[6-

[(7-nitro-2-1,3-benzoxadiazol-4-yl)amino] hexanoyl]-sn-glycero-3-

phospho-L-serine (ammonium salt)] were purchased from Avanti Polar Lipids (Alabaster, AL, USA). NBD-PS powder stocks were dissolved in 95% ethanol and diluted to 10 μM with Hank’s balanced salt solution with 15 mM MgCl_2_ and without phenol red (HBSS-15 mM MgCl_2_; Gibco). The isolated MEF cells were genotyped and cultured in DMEM high-glucose medium with 5 μg/ml tamoxifen for 48 hours. Cells with the genotypes of *Tmem30a* ^*loxp/loxp*^ were used as controls, and cells with the genotypes of *Tmem30a* ^*loxp/loxp*^ CAG-CreER were used as mutant cells. MEFs were seeded onto 60 mm-plates until they reached 80% confluency. Then, the cells were washed with PBS and treated with 0.25% trypsin. The cells were then suspended in PBS and centrifuged for 5 mins at 300 g followed by removal of the PBS buffer. The MEFs were then re-suspended with per-warmed 10 μM NBD-PS buffer in a 1.5-ml Eppendorf tube (Corning, NY, USA) and incubated for 15 minutes at 25 °C. Subsequently, the cells were centrifuged for 5 minutes at 300 g, and the supernatant was removed and immediately placed on ice. To quantify the NBD-lipids translocated into the inner leaflet of the plasma membrane, lipids from the outer leaflet were removed by back-extraction. This was done by adding ice-cold HBSS supplemented with 2% bovine serum albumin (Sigma, St Louis, MO, USA) to the cells for 10 minutes on ice, and then centrifuging the cells at 4 °C for 5 minutes at 300 g, and this process was repeated 3 times. Subsequently, the cells were suspended and subjected to FACS analysis.

### Annexin V staining of MEFs

MEF cells were seeded onto 60 mm-plates with a polylysine-treated coverslip until they reached 60% confluency. The cells were washed with PBS and stained with annexin V in the staining buffer supplied with the testing kit. The cells were then washed with PBS three times and counterstained with DAPI.

### Data Availability

All data generated or analysed during this study are included in this published article (and its supplemental information files).

## Electronic supplementary material

Supplementary data
