# Supplementary material for: Loss of Tmem30a leads to photoreceptor degeneration
Source: Sci Rep. 2017 Aug 24;7:9296. doi: 10.1038/s41598-017-09506-5 (PMC5571223; doi:10.1038/s41598-017-09506-5)
Supplement: Supplementary file 1 — Supplementary data [file 41598_2017_9506_MOESM1_ESM.pdf]

Loss of *Tmem30a* leads to photoreceptor degeneration

Lin Zhang[1,2]*, Yeming Yang[1,2]*, Shujin Li[1,4], Shanshan Zhang[1], Xiong Zhu[1], Zhengfu Tai[1,4], Mu Yang[1,4], Yuqing Liu[1,2], Xinzheng Guo[5], Bo Chen[5], Zhilin Jiang[1], Fang Lu[1,2#], Xianjun Zhu[1,2,3,4#]

*Contributed equally to this work

Supplemental data were presented as figure S1-13 and table S1 and S2.

Material and methods for supplemental data

RT-PCR

Tissue from 1–2 months old animals were dissected free and placed into RNAlater (Ambion) at room temperature. Total RNA was prepared from these tissues using TRIzol Reagent (Life Technologies) according to the manufacturer's instructions. RNA samples were treated with RNase-free DNaseI (Ambion) to remove genomic DNA and RNA concentration was determined using a NanoDrop (ND-1000) spectrophotometer. 3 μg RNA was reverse transcribed using random primers and the MessageSensor RT Kit (Ambion, TX, USA). The primers used for RT-PCR for mouse genes study were: *Atp8a2*, mAtp8a2F1=*5′*-GGAGCAGATCCTGGAGATTGACT-*3′*; mAtp8a2R1=*5′*- GGAGGTGTCTTCCTGCTGAG-*3′*; Atp8a1, Atp8a1-F, TGTGTGCTTGCCCGCTGGAC, Atp8a1-R, CACCCAGAAGACTCCAGAGC; *Atp11a*, Atp11a-F, CTGGCGGGTGTTCATTTACT-3', Atp11a-R, TGACAGTGAGCACCATCACA; *Atp11b*, Atp11b-F, TTTTGGGCTCCCAGAATATG-3', Atp11b-R, TTCTTCCAACAGACGCACAC; *Tmem30a,* Tmem30a-F, TGCCAACAGCATGTTTAATGA, Tmem30a-R, TTCGAGGCTCTCTTTTCCAG; *Tmem30b,* Tmem30b-F, AACGACTCCTTCTCGCTCTG, Tmem30b-R, CACGAAGTCCTGGTTGATGA; *Tmem30c*, Tmem30c-F, TTTCGGGAATCCAAGATCCAG, Tmem30C-R, CAGTCGGCGGTACAGTTTT; *beta-Actin*, mActinbF1=*5′*-AGCCATGTACGTAGCCATCC-*3′*; mActinbR1=*5′*-CGGCCAGCCAGGTCCAGAC-*3′*. PCR was performed using Taq (New England Biolabs, MA) and the PCR products were resolved on 3% agarose gels.

Long range PCR screening of Tmem30a KO first allele

Long range PCR were performed using specific primers to screen for positive

germline allele at both 5’ and 3’ arms. Primers used for long rang PCR were: Primers used for long-range PCR for 5’ arm are: Tmem30a-GF3, GAGGAAGCGGAAGTGTAAGTTACCAAG; Tmem30a-LAR3, CACAACGGGTTCTTCTGTTAGTCC. Primers used for long-range PCR for 3’ arm are: Tmem30a-GR3, GTGTGAAGTCAACGTCATTATCGGAGAATC; Tmem30a-RAF5, CACACCTCCCCCTGAACCTGAAAC. A 6.3 Kb fragment was amplified for 5’ arm for mouse 204-1. Mouse 1 6.6Kb fragment was also amplified for the same mouse, proving the presence of targeting alleles.

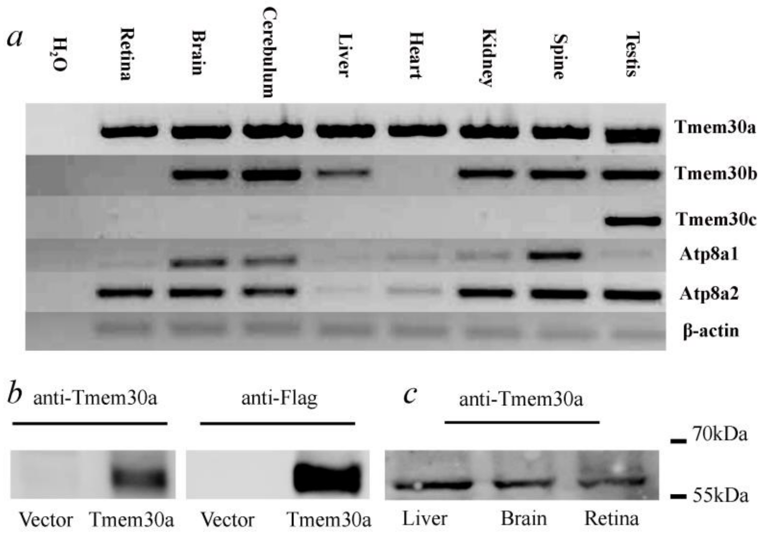

Figure S1. Tissue expression of *Tmem30a*. (A) RT-PCR analysis of *Tmem30a, Tmem30b, Tmem30c, Atp8a1,* and *Atp8a2* in various mouse tissues. *Tmem30a* is broadly expressed in the retina, cerebellum, liver, heart, kidney, spinal cord and testis. *Tmem30b* is expressed in the cerebellum, liver, kidney spinal cord and testis, while Tmem30c is only expressed in the testis. The phosphatidylserine flippase *Atp8a1* is expressed in the brain and spinal cord. Weak expression is observed for *Atp8a1* in the retina, heart and kidney. *Atp8a2* is expressed in the retina, cerebellum, heart, kidney, spinal cord and testis. Beta-actin was used as a control. (B) The TMEM30A monoclonal antibody specifically recognizes a 55-kDa protein. (C) TMEM30A was expressed in the liver, brain and retina.

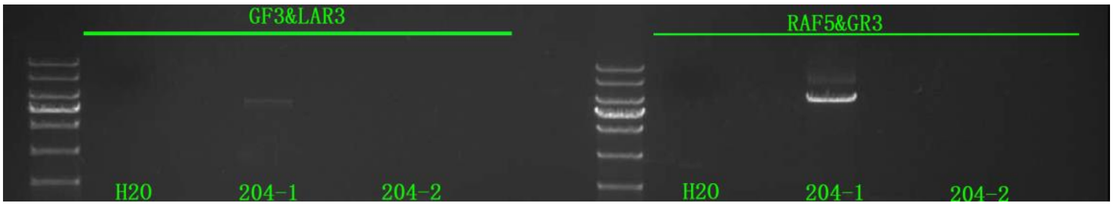

Figure S2. Long-range PCR screening of the *Tmem30a*-knockout first allele. Long-range PCR was performed using specific primers to screen for a positive germline allele at both the 5' and the 3' arms. A 6.3-Kb fragment was amplified for the 5' arm for mouse 204-1. Mouse 1 6.6-Kb fragment was also amplified for the same mouse, demonstrating the presence of the targeting alleles.

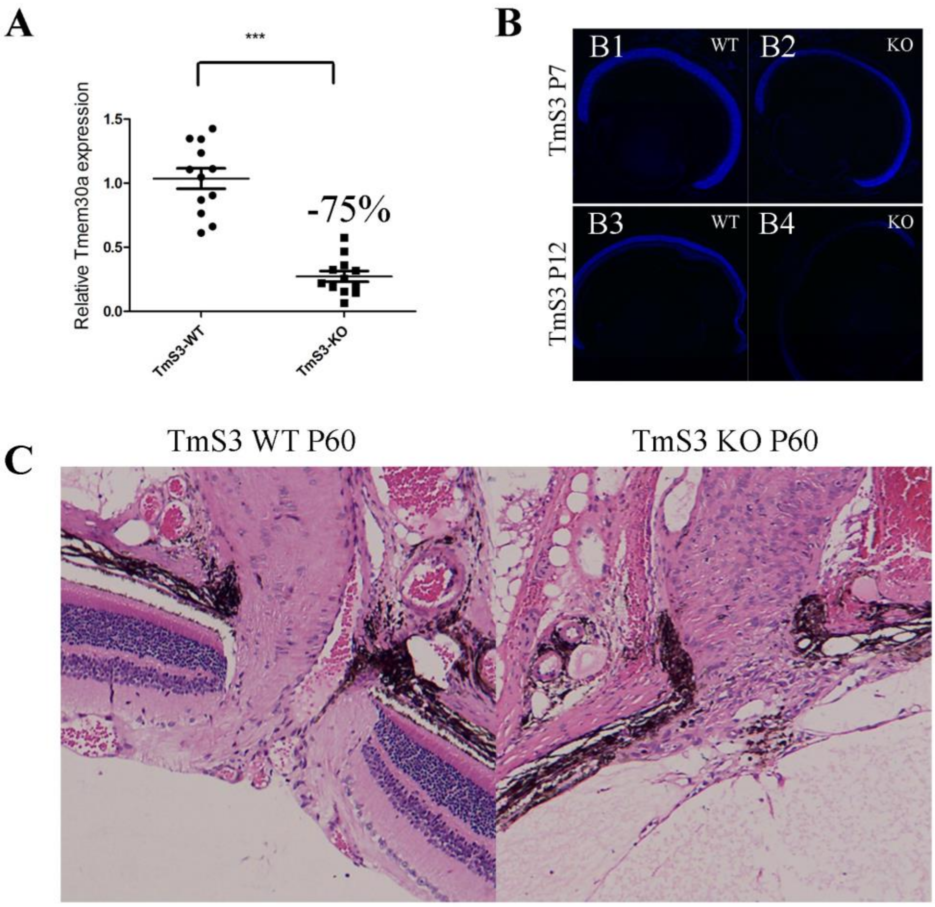

Figure S3. Phenotypes of the retinal knockout of *Tmem30a* Six-Cre mutant mice. (A) *Tmem30a* expression was greatly decreased, as revealed by Real-time-PCR with primers to amplify exon 1-2. (B) The loss of most retina cells in the mutant retina at P12. (C) H&E staining of wild-type and mutant retinas revealed a loss of retina tissues in the mutant. Only the optic nerve and trace amounts of the retina were left at the back of the eye.

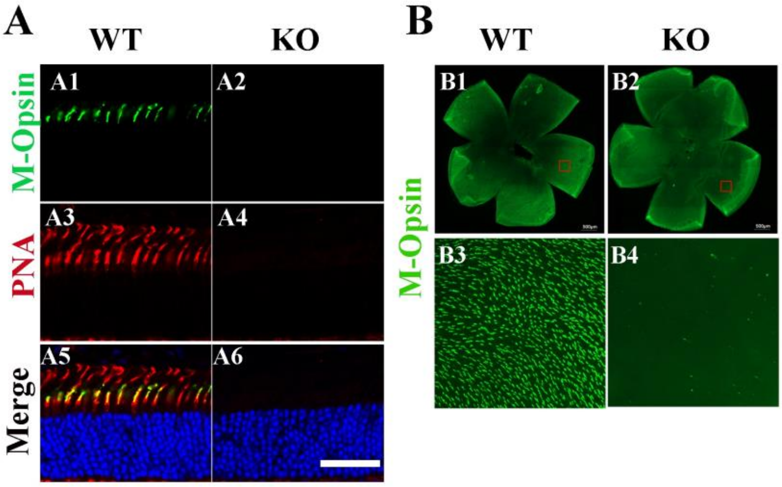

Figure S4. M-opsin expressing Cone cells were lost at P42 in *Tmem30a* cone-knockout mice. (A) Retina cryosections from P42 control (WT) and mutant (KO) littermates were immunostained with M-opsin (green), PNA (red) and DAPI (blue). In the WT retina, M-opsin localized to the outer segment of cone cells. However, in KO cone cells, no M-opsin expression was observed. (B) Retina whole mounts from P42 WT and KO littermates mice were immunostained with an M-opsin antibody to label M-opsin expressing cone cells. In the mutant retina, no M-opsin expressing cone cell left. Scale bar: A, 25μm; B，500μm.

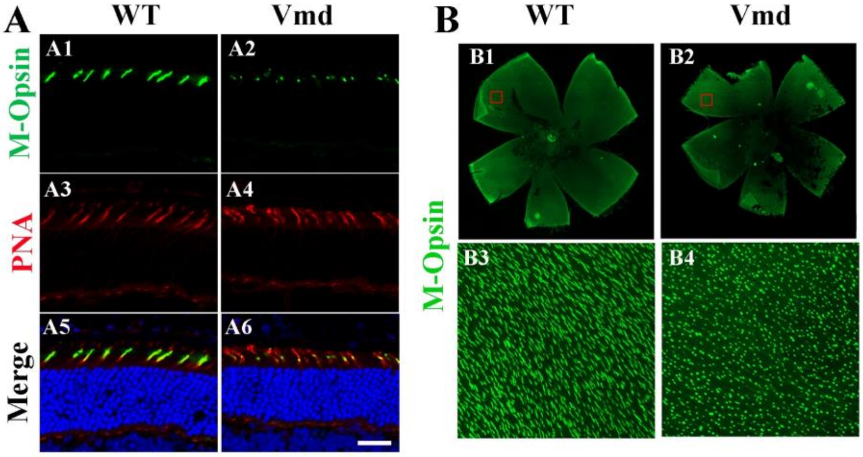

Figure S5. Cone opsin trafficking is impaired in *vmd* mice. (A) Retina cryosections from P30 control (WT) and mutant (*vmd*) littermates were immunostained with M-opsin (green), PNA (red) and DAPI (blue). M-opsin localized to the outer segment of cone cells. However, in *vmd*-mutant cone cells, the cone outer segment did not elongate properly, and M-opsin accumulated in dot-like structures. Scale bar: A, 25μm; B，500 μm.

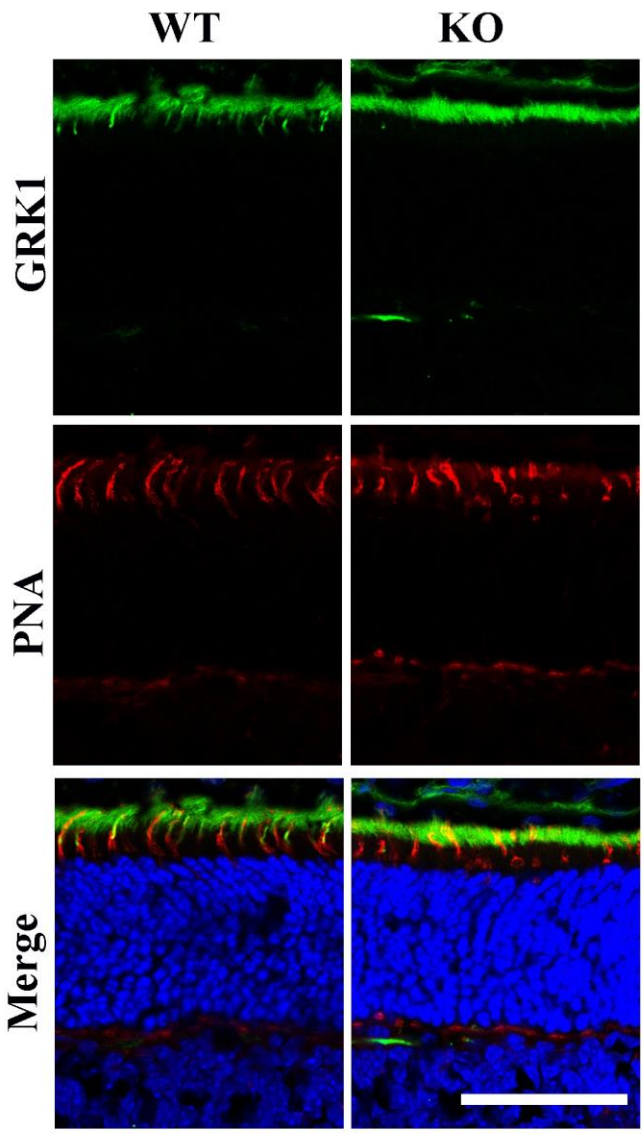

Figure S6. GRK1 expression is abolished in cones of *Tmem30a* cone knockout mice. Retina cryosections from P16 control (WT) and mutant (*Tmem30a*$^{loxp/loxp}$*, cone-Cre*) littermates were immunostained with GRK1 (green), PNA (red) and DAPI (blue). GRK1 localized to the outer segment of cone cells and rod cells in control retina. However, in KO cone cells, no GRK1 expression was observed (right panel). Scale bar: 25μm.

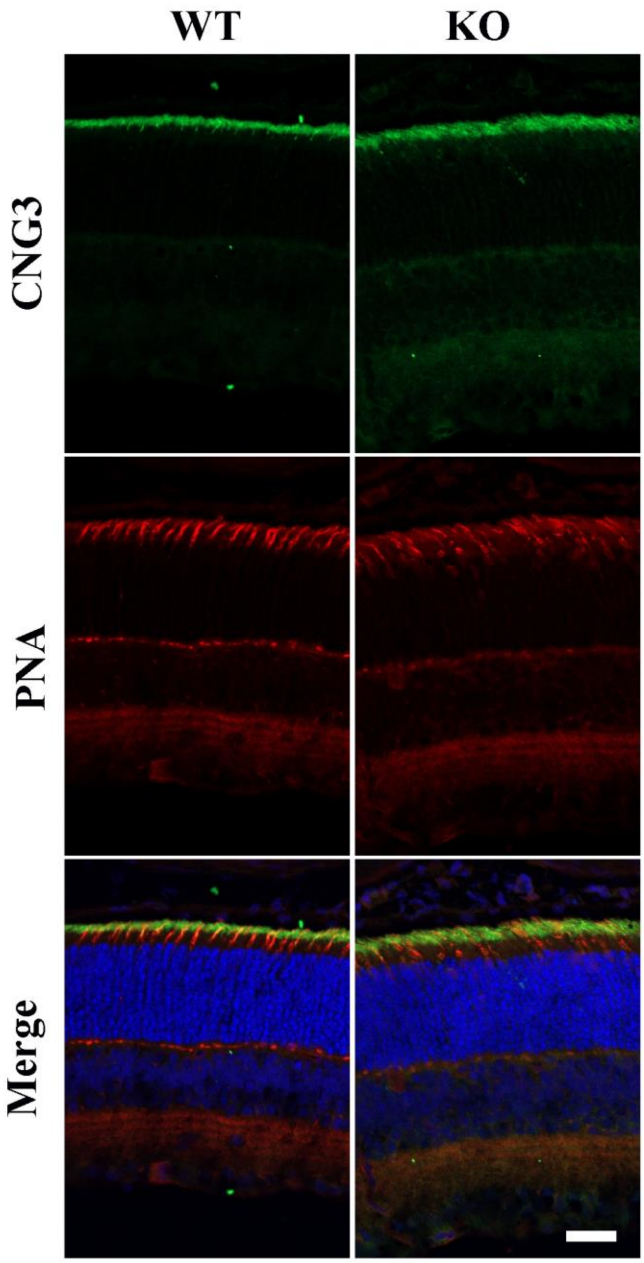

Figure S7. CNG3 expression is abolished in cones of *Tmem30a* cone knockout mice. Retina cryosections from P16 control (WT) and mutant (*Tmem30a*$^{loxp/loxp}$, *cone-Cre*) littermates were immunostained with CNG3 (green), PNA (red) and DAPI (blue). CNG3 localized to the outer segment of cone cells and rod cells in control retina. However, in KO cone cells, no CNG3 expression was observed (right panel). Scale bar: 25μm.

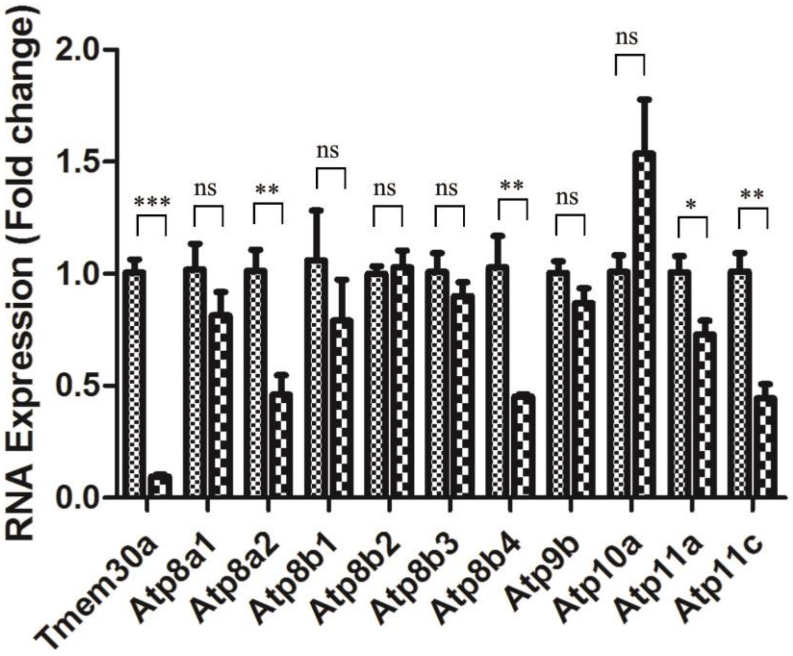

Figure S8. Real-time PCR analysis of P4-ATPase in wildtype and mutant MEFs. The expression level of *Tmem30a* in mutant MEFs was reduced to 10% of control cells. Compared to wildtype cells, the expression levels of *Atpa2*, *Atp8b4* and *Atp11a*, *Atp11c* were reduced in mutant MEFs. ***, p<0.001; **, p<0.01; * , p<0.05.

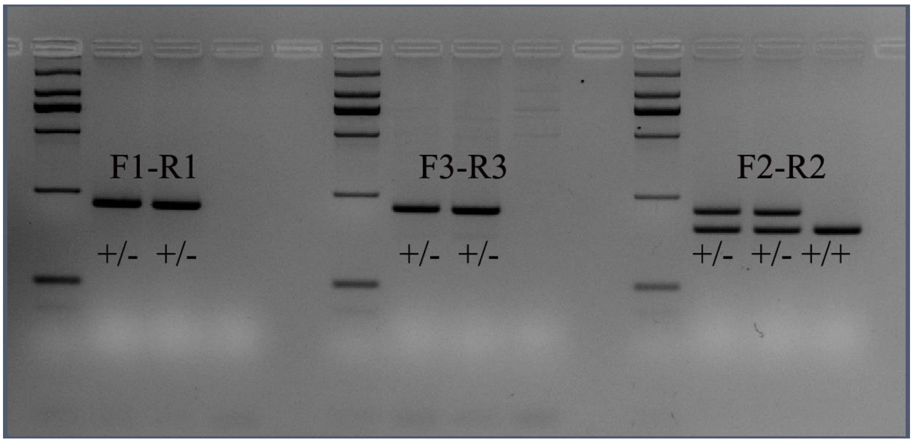

Figure S9. Full picture of panel in Figure 1B. Cropped image was used in Figure 1 to save space.

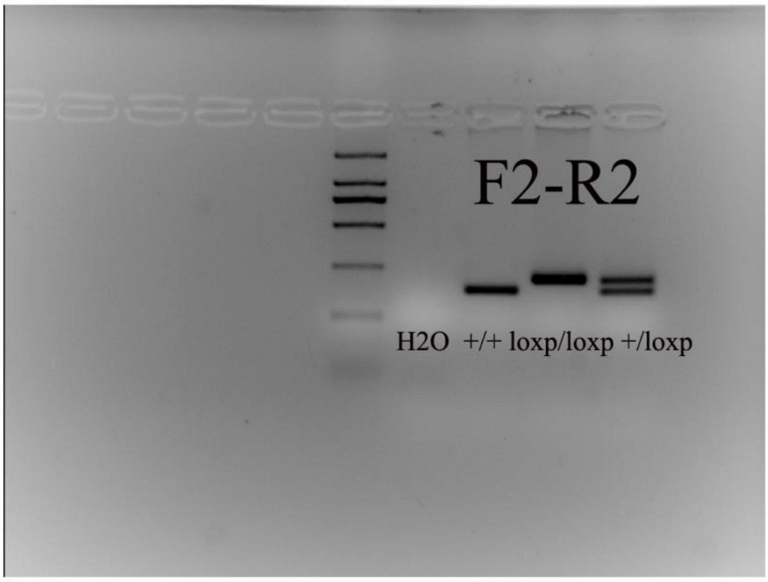

Figure S10. Full picture of panel in Figure 1C. Cropped image was used in Figure 1 to save space.

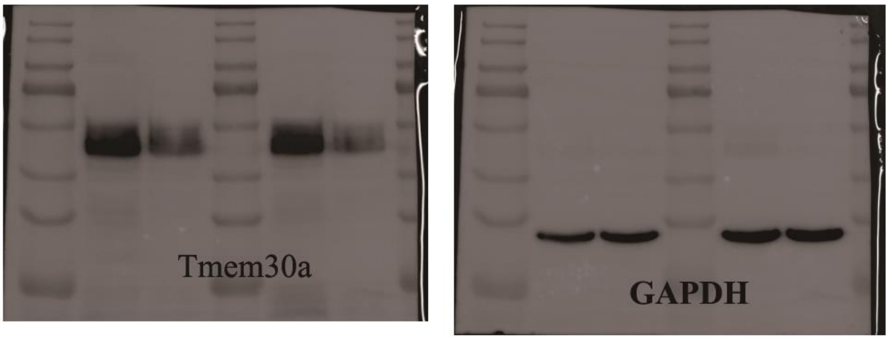

Figure S11. Full picture of panel in Figure 6B. Cropped image was used in Figure 6B to save space.

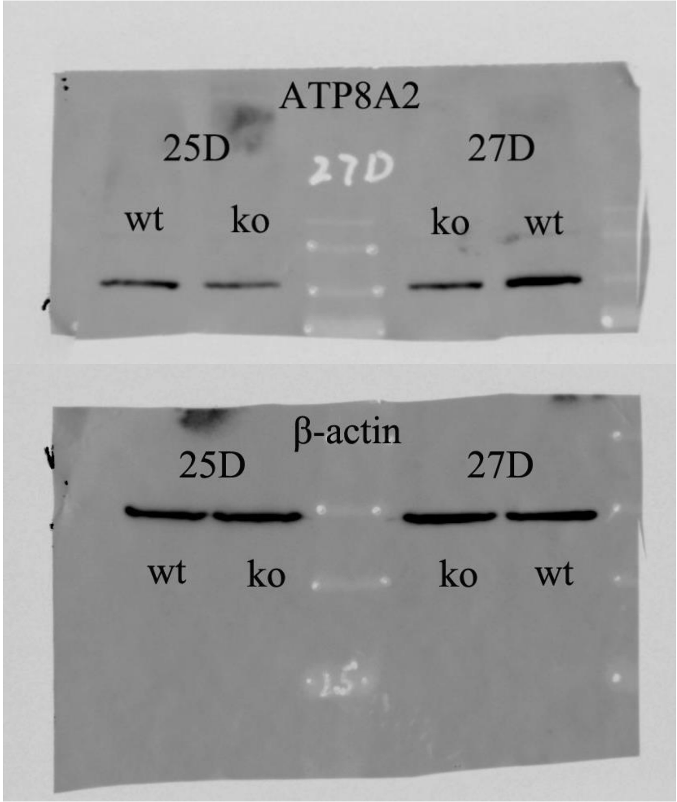

Figure S12. Full pictures of image panels in Figure 6C. Cropped gen pictures were used to save space.

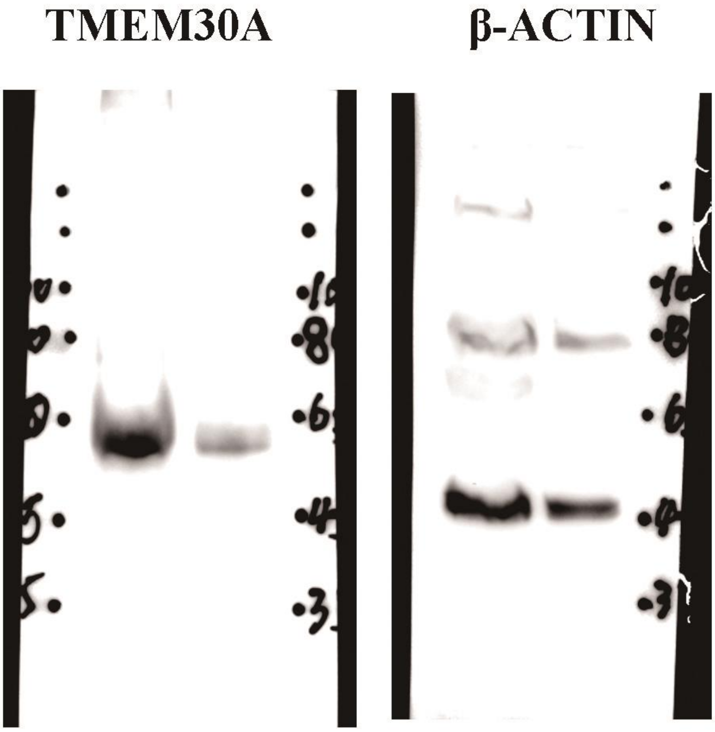

Figure S13. Full pictures of image panels in Figure 8A. Cropped gen pictures were used to save space.

Table S1. *Tmem30a* knockout intercross F2 genotype

	+/-	+/+	-/-
Quantity of offspring at P1	42	22	0
Percentage (%)	65.6	34.4	0
Mendelian ratio (%)	50	25	25

F1 genotype: Female: +/-; Male: +/-.

Table S2. *Tmem30a*$^{loxp/+}$, Nestin-Cre intercross F2 genotype

	+/ loxp	loxp/loxp	+/loxp, Tg	loxp/loxp,Tg
Quantity of offspring at P1	14	13	17	0
Percentage (%)	31.8	29.6	38.6	0
Expected mendelian ratio (%)	25	25	25	25

F1 genotype: Female: loxp/loxp; Male: +/loxp, Nestin-Cre
